# Reconstructing past changes in locus-specific recombination rates

**DOI:** 10.1186/1471-2156-14-11

**Published:** 2013-02-25

**Authors:** Murray P Cox, Barbara R Holland, Matthew C Wilkins, Jan Schmid

**Affiliations:** 1 Institute of Fundamental Sciences, Massey University, Private Bag 11 222, Palmerston North, 4442, New Zealand; 2 School of Mathematics and Physics, University of Tasmania, Hobart, Australia

**Keywords:** Recombination rate, Temporal, Reconstruction, Statistical inference

## Abstract

**Background:**

Recombination rates vary at the level of the species, population and individual. Now recognized as a transient feature of the genome, recombination rates at a given locus can change markedly over time. Existing inferential methods, predominantly based on linkage disequilibrium patterns, return a long-term average estimate of past recombination rates. Such estimates can be misleading, but no analytical framework to infer recombination rates that have changed over time is currently available.

**Results:**

We apply coalescent modeling in conjunction with a suite of summary statistics to show that the recombination history of a locus can be reconstructed from a time series of genetic samples. More usefully, we describe a new method, based on *n*-tuple dataset subsampling, to infer past changes in recombination rate from DNA sequences taken at a single time point. This subsampling strategy can correctly assign simulated loci to constant, increasing and decreasing recombination models with an accuracy of 84%.

**Conclusions:**

While providing an important stepping-stone to determining past recombination rates, *n*-tuple subsampling still exhibits a moderate error rate. Theoretical limitations indicated by coalescent theory suggest that highly accurate inference of past recombination rates will remain challenging. Nevertheless, we show for the first time that reconstructing historic recombination rates is possible in principle.

## Background

Meiotic recombination, whereby DNA variants are shuffled between homologous parental chromosomes, is a fundamental process in the evolution of genetic diversity. For many years poorly studied, the mechanisms and effects of recombination are now increasingly well understood [1]. We know that recombination rates are both heritable [2] and variable among individuals [3-7]. In other words, recombination is a Darwinian evolutionary system [8,9].

Recent studies have demonstrated convincingly that recombination rates at a given locus vary at the level of the species, population and individual. Comparisons between the chimpanzee and human genomes show poor correlation of both hotspot and background recombination rates at orthologous loci [10,11]. Similarly, recombination rates vary between human populations [11], not only at continental scales, but also between close geographical neighbors (e.g., French and Italians) [11]. Recombination rates even vary widely between individuals drawn from the same population [3-7]. The picture now emerging is one of an extremely dynamic recombination landscape [10], with transient recombination peaks and troughs across the human genome, overlaying the better-known evolutionary variation in DNA substitution rates [12].

How changes in recombination rate are controlled is less well understood [13]. At some loci, recombination events are determined by nucleotide variation within specific DNA sequence motifs (such as the degenerate 13-bp pattern recognized by *PRDM9*) [14,15]. These *cis*-mediated recombination events often show evidence of transmission distortion [16], where biased gene conversion preferentially favors one allelic variant that can rapidly reach fixation [17]. Related mechanisms may also act as a selective force to reduce recombination around functional genomic elements [18]. However, recombination rates at most loci seem to be mediated by *trans* factors [5], typically controlled by genes that coordinate DNA-protein interactions [19,20], or more generally, by regional chromatin remodeling [1,7]. These studies suggest that *trans*-mediated recombination processes dominate genome-scale recombination events and are not obviously under the influence of natural selection.

Recombination is typically detected either directly by gamete typing, or indirectly from linkage disequilibrium (LD) patterns [1]. Gamete typing surveys large numbers of recombination events within a single generation, and therefore provides an accurate (albeit costly) estimate of *contemporary* recombination rates. Conversely, statistical analysis of linkage disequilibrium patterns counts recombination events that have accrued over multiple generations, and therefore returns a long-term average estimate of *historic* recombination rates. Since recombination rates can change through time, contemporary and historic rate estimates need not agree. Regions of high recombination, as predicted from linkage disequilibrium, may be inactive when surveyed using gamete typing [21], a discrepancy that indicates the extinction of a previously high recombination region. Conversely, gamete typing may reveal regions of high recombination where none are suggested by linkage disequilibrium, thus indicating the birth of new high recombination loci [22].

The main point is that recombination rates at a genomic location can vary substantially through time. Although this fact is now widely appreciated [16], the manner in which recombination rates increase or decrease still remains completely unknown. Do changes in recombination rate occur rapidly, perhaps due to point mutations suddenly altering the action of the recombination machinery? Or are changes more gradual, occurring as regional nucleotide diversity mutates slowly over time? We currently lack any analytical framework to address these sorts of questions. Here, we determine that a suite of summary statistics can track changes in recombination rate through time. We extract temporal information about changing recombination rates, and describe some of the theoretical limitations that constrain this endeavor. More importantly, we develop a novel methodology based on *n*-tuple subsampling that has sufficient statistical power to reconstruct the recombination history of a genetic locus studied at a single time point. This approach is intended as a proof-of-concept that past changes in recombination rates can be reconstructed from contemporary data, even if reconstructing historic rates from empirical data remains challenging.

## Results

### Correlation and sensitivity of summary statistics

We first explored how different summary statistics respond to recombination events. The number of segregating sites *S* was used as a negative control because mean *S* does not vary with the recombination rate. We also assembled a suite of eight summary statistics that were designed specifically to detect recombination events – *R*_*min*_, *rmmg*, the number of haplotypes, haplotype diversity, Wall’s *B* and *Q*, Hudson’s *C* and *Z*_*nS*_. These summaries likely recognize different aspects of recombination, although the relationships between them have not been explored. Certainly none of these summaries capture the entire recombination profile of a genetic sample (i.e., they are not statistically sufficient).

**Figure 1:**
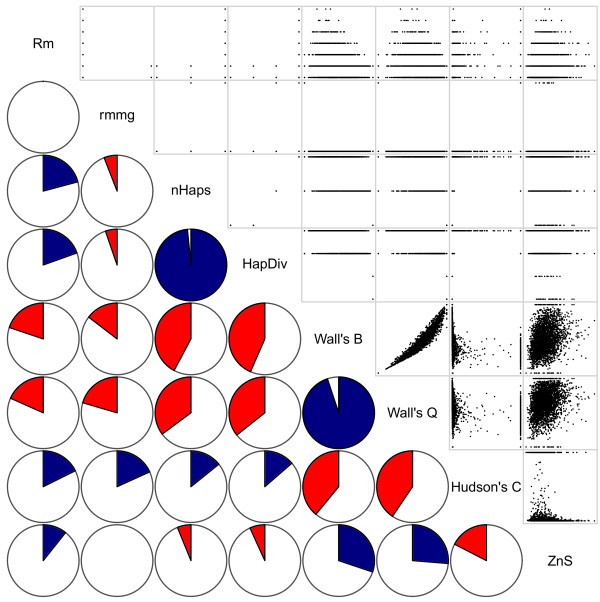
**Correlations between recombination summary statistics.** (Upper diagonal) Scatter plots show pairwise relationships among the summary statistics. (Lower diagonal) Pie charts show the magnitude of the correlation with blue and red indicating positive and negative values (e.g., Pearson’s *r* = −0.20 for Hudson’s *C* and *Z*_*nS*_). All non-zero correlations are statistically significant (*P* ≤ 0.05).

We studied the correlation matrix between summary statistics using an equal mix of datasets with linearly increasing, decreasing and constant recombination rates. *A priori*, we might expect that many of the summaries detect similar aspects of the overall recombination signal. Indeed, pairwise comparisons indicated that nearly all the summaries were correlated, albeit to different extents (*r* values range from 0 to 0.982, mean of 0.351) (Figure 1). The smallest correlations involved *rmmg*, a conservative lower bound on the minimum number of recombination events *R*_*min*_, which showed little variation among datasets under the conditions modeled here. None of the summary statistics were perfectly correlated, thus emphasizing that multiple summaries are needed to capture different aspects of the recombination profile.

**Figure 2:**
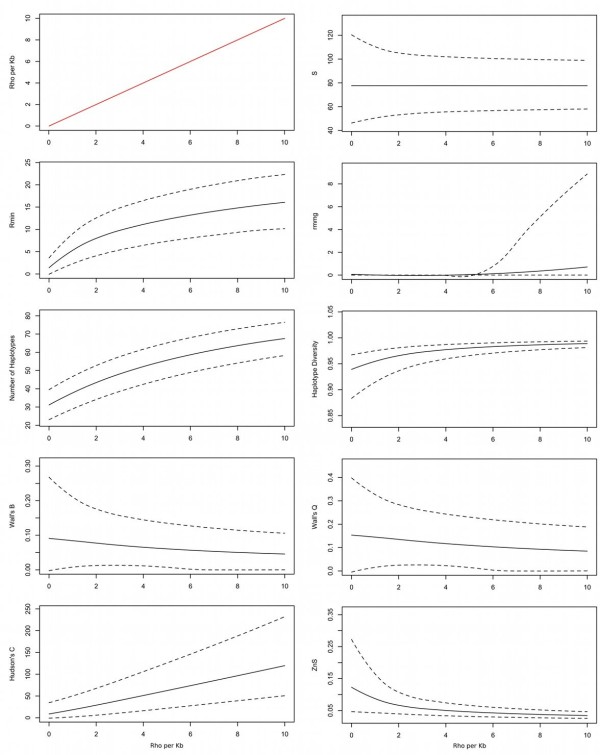
**Sensitivity of summary statistics to different constant recombination rates.** Black lines show the mean (solid) and 95% confidence intervals (dotted) of summary statistic values. The red line indicates different constant recombination rates (*ρ* per kb). Note that summary statistics mostly vary nonlinearly with linear change in recombination rates.

To determine how these summaries respond to different recombination rates, we simulated genetic data under a wide range of constant recombination values (0–10 *ρ*/kb) (Figure 2). *S* is shown as a negative control because its mean is invariant to the recombination rate (Figure 2, upper right). Most summary statistics varied nonlinearly across this linear range of recombination values. It follows that the usefulness of any individual summary may change with the underlying recombination rate, but in different ways. Therefore, a combination of some or all of these summaries may be more sensitive for detecting different recombination rates than any one of them alone.

### Tracking changing recombination rates using time series data

It is less obvious how summary statistics might covary with recombination rates that change over time. To explore this process, we generated coalescent simulations where recombination rates were allowed to vary over many generations. Genetic datasets were simulated using coalescent software [23] modified to allow recombination rates to change through time. We simulated data for a human-like deme: 10^4^ replicates of 10-kb autosomal sequences were drawn from a constant sized population (*N*_*e*_ = 10^4^) [24-26] with a mutation rate, *μ*, and average recombination rate, *r*, of 3.75 × 10^-8^ events/bp/generation [27]. These rates were chosen to mimic regions of very strong recombination in real human populations [11]. The recombination rate was either held constant, or allowed to vary linearly, exponentially or logistically through time for 10^4^ generations (cf. [24,26]). The total amount of recombination was constrained to be identical for all models; only its distribution through time was altered.

**Figure 3:**
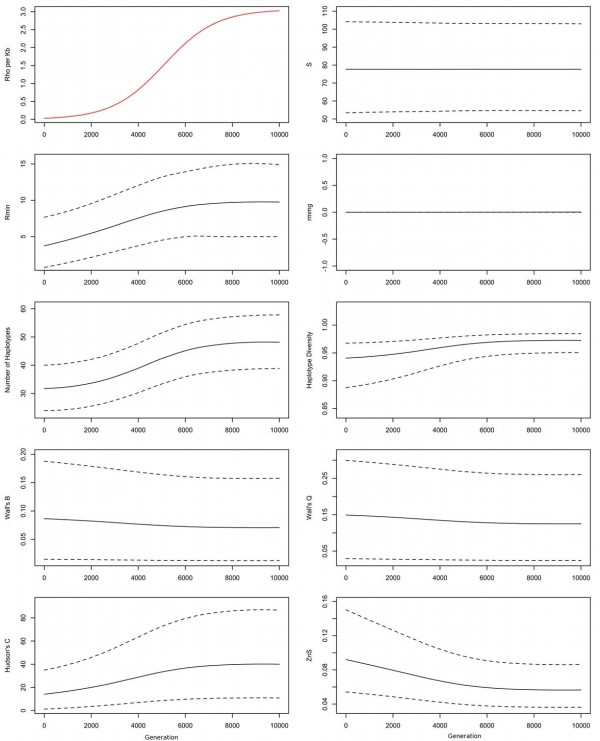
**Response of summary statistics to recombination rates changing logistically over time.** Black lines show mean (solid) and 95% confidence intervals (dotted) of summary statistic values. The red line indicates how the recombination rate changes over time (*ρ* per kb). Note the lag between changes in the recombination rate and changes in summary statistic values. Compare responses for constant recombination, as well as linearly, exponentially and logistically increasing and decreasing recombination rates, in Additional file 1: Figure S1, Additional file 2: Figure S2, Additional file 3: Figure S3, Additional file 4: Figure S4, Additional file 5: Figure S5, Additional file 6: Figure S6 and Additional file 7: Figure S7.

A representative example illustrating a logistic decline in recombination rates towards the present is presented in Figure 3. Corresponding plots for constant recombination, together with recombination rates increasing and decreasing linearly, exponentially and logistically, are presented in Additional file 1: Figure S1, Additional file 2: Figure S2, Additional file 3: Figure S3, Additional file 4: Figure S4, Additional file 5: Figure S5, Additional file 6: Figure S6 and Additional file 7: Figure S7.

Most of the summary statistics tracked the changing recombination profile, albeit with notable differences in accuracy. The variance of many summaries altered with the recombination rate, thus suggesting that different summaries have greatest power to estimate recombination rates at different times. This reinforces the view that using a combination of summary statistics should maximize statistical power, although a simple linear combination may not necessarily be optimal.

Note too that summary values typically lagged changes in the recombination rate. Genetic variation observed in the present was actually laid down in the (sometimes very distant) past [28]. As recombination rates change, it takes time before this change is reflected in the genetic record. This lag effect is perhaps best illustrated in the plot showing recombination rates increasing exponentially into the past (Additional file 5: Figure S5). Although the recombination rate drops quickly, the summaries change far more slowly. Even after the recombination rate falls near zero, existing lineages still retain the signal of recombination events that occurred further back in the past. Only as these recombined lineages are lost through genetic drift is the new low recombination rate finally reflected in the summaries. This time lag places important constraints on the resolution with which recombination rates that have changed through time can be reconstructed.

### Reconstructing past recombination rates from data taken at a single time point

Tracking variable recombination rates using time series data may be feasible for some fast evolving systems (e.g., exploring the loss of sexual competency in yeast), but it is not practical for long-lived organisms like humans. To explore whether past recombination rates can be reconstructed from genetic data taken at a single time point, we developed a novel bootstrapping methodology that we call *n-*tuple subsampling.

Mutations occur randomly through time. In any given dataset, some polymorphisms will be old and most modern lineages will carry them. Others will be young, and will therefore be found in only one or two individuals. By determining whether recombination events affect young or old polymorphisms, we can theoretically obtain snapshots of recombination rates through time.

**Figure 4:**
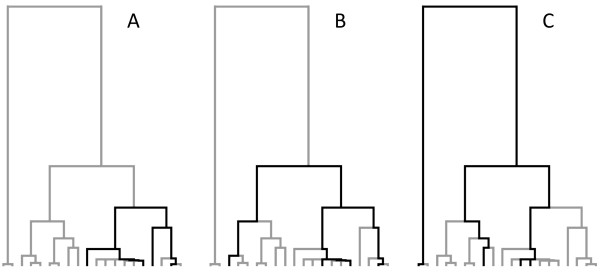
**Variable ages of quartets.** Randomly selected quartets (black lines) capture information about (**A**) young, (**B**) medium and (**C**) old time depths. For visual clarity, quartets are shown on a non-recombining genealogy, but the principle holds equally for ancestral recombination graphs.

This concept is best shown graphically (Figure 4). Imagine we repeatedly subsample a group of four lineages (i.e., an *n*-tuple of four, or a ‘quartet’) from a given dataset. These individuals may be closely related (Figure 4A), in which case they contain information about recombination rates in the recent past. Alternately, the individuals may be only distantly related (Figure 4C), in which case they may carry both young and old recombination events. By repeatedly resampling the dataset, the recombination rate at different times can be inferred. We emphasize that *n-*tuple subsampling has a natural confound. Young *n-*tuples carry information about recent recombination rates, but old quartets contain a mix of information about old and recent recombination events. The statistical power of this approach is therefore unclear and we explore this issue in detail below.

The use of resampling methods, such as the bootstrap and jackknife [29], to estimate sampling distributions is widespread in statistics in general, but especially so in evolutionary biology [30]. More recently, interest has grown in so-called ensemble methods (also referred to as ‘bagging and boosting’) that seek to improve overall accuracy by combining the predictions of many weak classifiers, each of which is shown a slightly perturbed version of the data [31]. The following implementation of *n*-tuple subsampling differs from the ensemble method as we use only a single classifier. However, it is a related concept in that the classifier is shown many subsets of the data, which in our case is used to develop combinations of summary statistics that capture information about recombination rates over different time periods.

To ascertain whether *n*-tuple subsampling has sufficient power to estimate past recombination rates, we created a test system where datasets derived from only one of three recombination models: recombination that is constant, linearly increasing or linearly decreasing through time (10^4^ datasets each) (Additional file 1: Figure S1, Additional file 2: Figure S2 and Additional file 3: Figure S3). 10^3^ quartets were generated for each dataset, and the suite of summary statistics was calculated for each subsample. The mean, variance and maximum of these summary statistic distributions were recorded.

Although powerful Bayesian and maximum likelihood methods have been developed to perform inference on such datasets [32,33], these approaches are extremely computationally expensive (i.e., tens to thousands of CPU hours to analyze a single dataset) [25,26,34,35]. As we needed to run power analyses for thousands of test cases, a more pragmatic analytical framework was necessarily adopted (cf. [36]). We instead employed discriminant analysis [37], a routine statistical technique for data classification, with jackknife cross-validation to evaluate the accuracy of the classifier. Discriminant analysis infers the combination of weighted summaries (i.e., the optimal transform) that best distinguishes the recombination models. In jackknife cross-validation, model labels were removed, and each blinded dataset was instead assigned to a recombination model using the discriminant function. Assignment accuracy was calculated by determining the proportion of datasets that were assigned to the correct recombination model (i.e., datasets derived under a constant recombination model should be assigned back to the constant model). Both linear discriminant analysis (LDA) and quadratic discriminant analysis (QDA) were tested. These related methods respond differently to unequal covariance among models, as well as different sizes of sample and training sets [38]. LDA proved to return more accurate classifications in this instance.

LDA was performed on all datasets from all three recombination models. Each dataset was sequentially excluded, the optimal transform inferred by LDA was applied, and each dataset was reassigned back to a recombination model. As we have three models, assignment rates of one-third are expected just by chance. Assignment rates approaching one indicate increasingly accurate assignments.

Table 1 shows observed assignment rates. Because the mean of *S* is invariant to recombination (Figure 2, upper right), assignments using *S* alone are presented as a negative control. As expected, the mean, variance or maximum number of segregating sites performed no better than chance. The best individual summary, the mean number of haplotypes, was much more accurate (60%). The best result was obtained by combining the mean, variance and maximum values of all summaries (68%), although 32% of datasets were still placed incorrectly.

**Table 1:** Assignment accuracy using linear discriminant analysis on quartets

	***Mean***	***Variance***	***Maximum***	***Combined***
***S***	0.36	0.32	0.36	
***R***_***min***_	0.43	0.45	0.38	
***rmmg***	0.43	0.43	0.45	
**nHaps**	0.60	0.59	0.33	
**HapDiv**	0.59	0.57	0.33	
**Wall’s *B***	0.41	0.47	0.34	
**Wall’s *Q***	0.40	0.37	0.33	
**Hudson’s *C***	0.43	0.42	0.33	
***Z***_***nS***_	0.48	0.34	0.48	
**All unscaled**	0.66	0.65	0.52	0.68
***S *x *R***_***min***_	0.36	0.36	0.34	
***S *x *rmmg***	0.43	0.41	0.45	
***S *x nHaps**	0.32	0.36	0.36	
***S *x HapDiv**	0.32	0.36	0.36	
***S *x Wall’s *B***	0.37	0.33	0.31	
***S *x Wall’s *Q***	0.37	0.34	0.32	
***S *x Hudson’s *C***	0.42	0.37	0.37	
***S *x *Z***_***nS***_	0.42	0.34	0.43	
**All scaled**	0.64	0.59	0.53	0.67
**All combined**				0.71

Proportions of datasets assigned correctly to constant, linearly increasing and linearly decreasing recombination models using jackknife cross-validation. Values of one-third indicate assignments no better than chance; values approaching one indicate improving assignment rates. 10^4^ datasets consisting of 10-kb of sequence for 100 individuals were generated under each model. Assignments were made using the mean, variance and maximum value of summary statistics for 10^3^ quartets for each dataset.

### Scaling subsamples by *n*-tuple Age

These assignments were obtained using information about the amount of recombination in each *n*-tuple, but not its age. When recombination rates change over time, the amount of recombination and the age of each *n*-tuple should be correlated. We would therefore prefer to use summaries that capture information about both factors simultaneously. To develop such summaries, we scaled the recombination summaries by *S*, which is a robust proxy for *n*-tuple age (Additional file 8). Pairwise correlations indicated that most scaled summaries are positively correlated (*r* values from 0.055 to 1, mean of 0.436) (Additional file 9: Figure S9). The cross-validation test was repeated, and surprisingly, the scaled summaries often performed more poorly than their unscaled versions. Nevertheless, using the mean, variance and maximum values of both the scaled and unscaled summaries returned the best overall result (71% correct assignment). This suggests two key conclusions. First, genetic datasets do record retrievable information about past changes in recombination rate. Second, scaled and unscaled recombination summaries do capture very slightly different information from the recombination profile.

Coalescent theory tells us that the power to detect recombination events should decline exponentially into the past (see details later). Therefore, the linearly increasing and decreasing models are mostly dominated by low and high recombination rates, respectively, while the constant model is intermediate. We were concerned that our cross-validation test might simply be detecting low, medium and high recombination rates rather than distinguishing constant recombination from recombination rates that change through time. We therefore repeated the cross-validation test with four recombination models: constant high, constant low, linearly increasing and linearly decreasing recombination rates. Assignment accuracy was only slightly lower than for the three-model test (64% vs 71%). We conclude that *n*-tuple subsampling can distinguish changing recombination from constant recombination, as well as rates that increase or decrease through time.

### Effect of *n*-tuple size on classification accuracy

**Figure 5:**
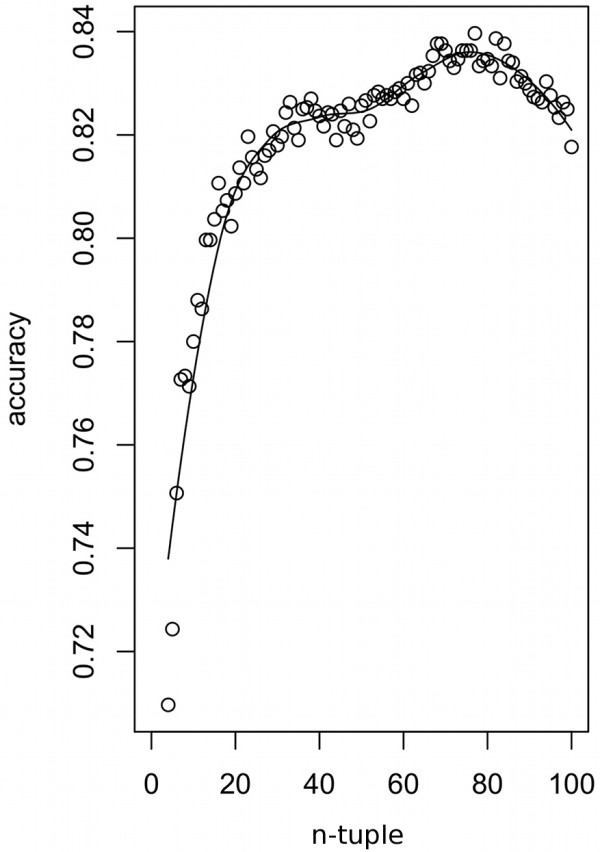
**Effect of subsample (*n-*tuple) size on assignment accuracy.** Subsample sizes range from 4 to the sample size (*n* = 100). The curve shows a local regression through the data points.

Thus far, *n*-tuple subsampling has been performed using just four sequences (a quartet). Quartets have found many uses in phylogenetics because this is the minimum number required for unrooted trees to possess distinguishable topologies. However, quartets may not be optimal for reconstructing past recombination rates within a population. We therefore varied the subsample size from 4 to the total sample size, 4 ≤ *n* ≤ 100 (Figure 5). The three-model system was used, and the optimal LDA transform was recalculated for each value of *n*.

As before, assignment rates started at 71% for quartets, initially improved with increasing *n*, before declining again as the subsample size approached the total sample size. The best assignment accuracy (84%) was obtained with an *n*-tuple of size 77. While subsampling a large proportion of the dataset seemed to be most informative under this model system, optimal subsample size appears to vary from dataset to dataset in practice. When recombination rates are low, recombination events are recorded by few lineages and subsampling a greater proportion of the dataset improves detection of these rare occurrences. However, we can only detect recombination rates from their effects on DNA polymorphisms, so recombination events will often pass undetected if nucleotide diversity is low. Both low recombination rates and low genetic diversity therefore favor larger subsamples, while smaller subsamples are preferable for highly diverse or recombination-rich regions. Apart from these general guidelines, it seems that optimal subsample sizes must be determined empirically for each dataset.

Assignment accuracy was maximized at 84% across all analyses performed here. Although considerably better than chance, the error rate is still moderate. Because power levels are relatively modest, reconstructing historic recombination rates for real genomic loci is expected to remain difficult even when *n*-tuple subsampling is employed. The highly constrained testing environment used here (e.g., a simple and perfectly known demography) emphasizes this point. In practice, complex demographic processes can produce patterns of genetic variation that might otherwise be attributed to processes of recombination [39]. Still, *n*-tuple subsampling is directly amenable to statistical methods that infer model likelihoods by simulating data across a parameter space (e.g., approximate Bayesian computation [32,33]). Like *n*-tuple subsampling, these methods typically employ a suite of summary statistics, and because they are based on Monte Carlo simulation, they can readily be modified to accommodation the novel bootstrapping process that we propose.

## Discussion

We show that information about past changes in recombination rate can be extracted from genomic data using a suite of summary statistics coupled with lineage subsampling to provide proxy information about recombination events at different time depths. Simulated datasets can be correctly assigned to different models of historic recombination with high accuracy (84%).

**Figure 6:**
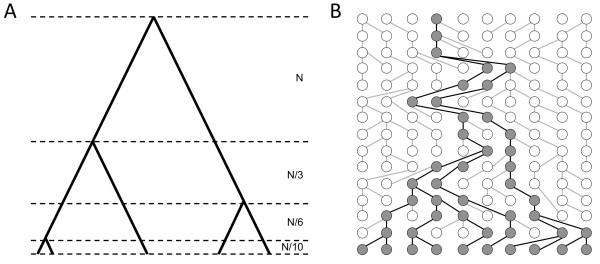
**Relationship between time and number of lineages under the coalescent.** (**A**) Expected coalescent times for 2–5 lineages in units of *N* generations for a haploid locus (2 *N* generations for autosomal loci). (**B**) Representative coalescent genealogy. Note that many lineages exist to record events in the recent past, while few lineages remain to represent older time points. Only recombination involving an extant lineage (shaded points) can be observed today. The probability that recombination involves an extant lineage is high in the present, where many shaded points exist, but declines exponentially into the past, where shaded points are scarce.

Why is the power of *n-*tuple subsampling not greater? Coalescent theory suggests several possible reasons. The coalescent describes how pairs of lineages sequentially share a common parent and merge (“coalesce”) until only one ancestral lineage remains. This process is analogous to genetic drift, where lineages are lost by chance over time. The key point is that individuals existing today are represented by fewer and fewer ancestral lineages moving backwards into the past (Figure 6).

Put more formally, the coalescent times *T*_*i*_ of *m* sampled lineages are exponentially distributed with mean and variance [40-42].

(1) $$E\left[T_{i}\right]=\frac{2}{i\left(i-1\right)}\;\text{for}\;i=2,\ldots ,m$$

(2) $$\mathrm{Var}\left[T_{i}\right]={\left(\frac{2}{i\left(i-1\right)}\right)}^{2}$$

In the sampling limit *m* → ∞, this exponential process implies that coalescence of the final two lineages (*T*_*2*_) takes, on average, half the time to the most recent common ancestor of the sampled dataset (Figure 6A). While these equations only hold for constant sized populations, the general principle is true for most demographic scenarios of relevance to human history. Many lineages record the recent history of a locus, but older time depths are represented by fewer and fewer lineages that still exist today. Indeed, only two lineages will be present for approximately half the total age of the tree. These rootward branches are expected to provide most of the signal for changes in historic recombination rates. However, recombination events from distant times can only be detected in the modern dataset if they directly affect the few old lineages that survive to the present (Figure 6B). Information about historic recombination events therefore declines exponentially into the past.

Further, there is a high probability of observing these two deepest branches, even with very small subsample sizes [43,44].

(3) $$P\left(\text{root}|n\right)=\frac{n-1}{n+1}$$

Given four randomly chosen subsamples (i.e., a quartet), *P*(root | *n* = 4) = 3/5 = 0.60. Even with just four quartets, the probability that the oldest two branches are not observed is less than 5%. This equation gives the average expectation across all trees, but the general principle holds even for highly unbalanced trees. At the *RRM2P4* locus, which putatively introgressed from archaic hominins into modern humans, 232 individuals are observed on one side of the tree’s basal split, while only 21 are found on the other [35]. The probability of observing the root with a single quartet is still high, *P*(root | *n* = 4) = 0.29. Importantly, all *n-*tuples that sample these two oldest branches return exactly the same information about these oldest recombination events (Figure 4C). It follows that simply observing a larger number of *n-*tuples is not sufficient to obtain more information about recombination in the oldest half of the ancestral recombination graph. Conversely, young quartets offer many possible sampling permutations (Figure 4A), and each of these can potentially provide independent information about recent recombination events. All datasets therefore record more information about recent recombination rates, while power to detect old recombination events declines exponentially backwards into the past.

## Conclusions

A natural limit places important constraints on our ability to reconstruct past changes in recombination rates. If the change occurred recently, sufficient extant lineages may still record the event, and *n*-tuple subsampling is likely to be an informative technique. Moving further in time from the change, the power to reconstruct the recombination profile decreases exponentially. If the change occurs beyond the coalescent (i.e., the most recent common ancestor of the dataset), it obviously cannot be reconstructed at all. Extremely detailed changes in past recombination profiles, particularly for more distant events or complex genomic loci, will remain challenging. However, we show that *n*-tuple subsampling does have sufficient power to reconstruct some aspects of past changes in recombination rates, especially for relatively recent events.

## Methods

### Simulations

The coalescent simulation software *ms *[23] was modified to allow recombination rates to change through time. The C source code of the resulting program, *ms_recomb*, is available from the authors on request. Simulations focus on the most common *trans*-mediated recombination events, and we therefore model changes in recombination without selection (i.e., no transmission distortion).

Genetic datasets were simulated using Kingman’s *n*-coalescent [40,41]. To ground the simulations in a realistic framework, model parameters were chosen to reflect biologically meaningful values for humans. We purposely simulated a generic human-like deme rather than any specific population. Simulations were generated for a single Wright-Fisher deme with a constant effective population size (*N*_*e*_ = 10^4^) (i.e., the estimated global effective population size of modern humans) [24-26]. A sample of 10-kb autosomal sequences was simulated for 100 individuals with a mutation rate, *μ*, and average recombination rate, *r*, of 3.75 × 10^-8^ events/bp/generation [27]. Unless otherwise noted, this process was iterated 10^4^ times for each model.

The recombination rate was either held constant, or allowed to vary linearly, exponentially or logistically through time for 10^4^ generations (cf.[24,26]). Linear rates were incremented by the reciprocal of the generation units per generation, exponential rates were fitted to a curve with *λ* = 5 × 10^-4^, and logistic rates were fitted to a curve with *K* = 100, *N* = 1 and *r* = 9 × 10^-4^. (Note that these curves are for exploratory purposes only. They are not intended to represent real rates of change in human populations). The total amount of recombination was constrained so as to be identical for all models, but was apportioned through time according to the constant, linear, exponential and logistic distributions described above. Overall population recombination rates (i.e., *ρ* = 4*N*_*e*_*r* = 15) were chosen to mimic regions of very strong recombination in real human groups [11]. Low and high constant rates were defined as 15% and 85% of the maximum rate under the corresponding linear models.

To infer past recombination rates, samples were taken at a single time point and surveyed using *n*-tuple subsampling (see main text for details). To determine how summary statistics respond to changing recombination rates, variation in summary statistics was tracked over a time span of 10^4^ generations by taking 10^4^ independent coalescent simulations at each of 500 20-generation intervals.

### Summary statistics

Summary statistics were calculated using functions from the libsequence library [45]. The C++ source code of the resulting program, *msstats_recomb*, is available from the authors on request. The number of segregating sites, *S*, controls for the population mutation rate *θ* (= 4*N*_*e*_*μ*) and summarizes the total length of the genealogy [46]. A suite of eight additional summary statistics was employed to capture different aspects of the recombination profile: *R*_*min*_, the minimum number of recombination events calculated from observed four-gamete violations [47]; *rmmg*, a conservative lower bound on *R*_*min*_ proposed by Myers and Griffiths (equation four in [48]); *nHaps*, the number of observed unique sequence haplotypes; *HapDiv*, the haplotype diversity, expected heterozygosity, or probability that two sequences chosen randomly from the sample are different [49]; Wall’s *B* and *Q*, variant estimators of the number of congruent polymorphic sites (i.e., segregating sites in complete linkage disequilibrium) [50]; Hudson’s *C*, an estimator of the population recombination rate *ρ* (= 4*N*_*e*_*r*) estimated from the variance of pairwise sequence differences [51]; and *Z*_*nS*_, the mean pairwise *r*^*2*^ estimate of linkage disequilibrium across all polymorphic sites [52].

### Statistics

Correlations between scaled and unscaled summary statistics, and discriminant analyses, were calculated using the statistical software *R*[53]. Local regressions were performed using a polynomial of degree 2, a smoothed-particle hydrodynamics (SPH) kernel, and a 50% nearest neighbor bandwidth with a 10% constant component.

## Competing interests

The authors declare that they have no competing interests.

## Authors’ contributions

MPC, BRH, MCW and JS conceived and designed the experiments. MPC performed the experiments. MPC, BRH and JS analyzed the data. MPC drafted the manuscript. All authors have read and approved the final manuscript.

## Supplementary Material

Additional 1: Figure S1 Response of summary statistics to constant recombination rates. Click here for file

Additional 2: Figure S2 Response of summary statistics to recombination rates decreasing linearly. Click here for file

Additional 3: Figure S3 Response of summary statistics to recombination rates increasing linearly. Click here for file

Additional 4: Figure S4 Response of summary statistics to recombination rates decreasing exponentially. Click here for file

Additional 5: Figure S5 Response of summary statistics to recombination rates increasing exponentially. Click here for file

Additional 6: Figure S6 Response of summary statistics to recombination rates decreasing logistically. Click here for file

Additional 7: Figure S7 Response of summary statistics to recombination rates increasing logistically. (PDF 279 kb) Click here for file

Additional 8 **Determining the optimal scaling factor to capture *n***-tuple age. Click here for file

Additional 9: Figure S8 Correlations between recombination summary statistics scaled by *S*. Click here for file
