# Supplementary material for: Reconstructing past changes in locus-specific recombination rates
Source: BMC Genet. 2013 Feb 25;14:11. doi: 10.1186/1471-2156-14-11 (PMC3605148; doi:10.1186/1471-2156-14-11)
Supplement: Additional 1: Figure S1 — Response of summary statistics to constant recombination rates. [file 1471-2156-14-11-S1.pdf]

Additional Figure 1

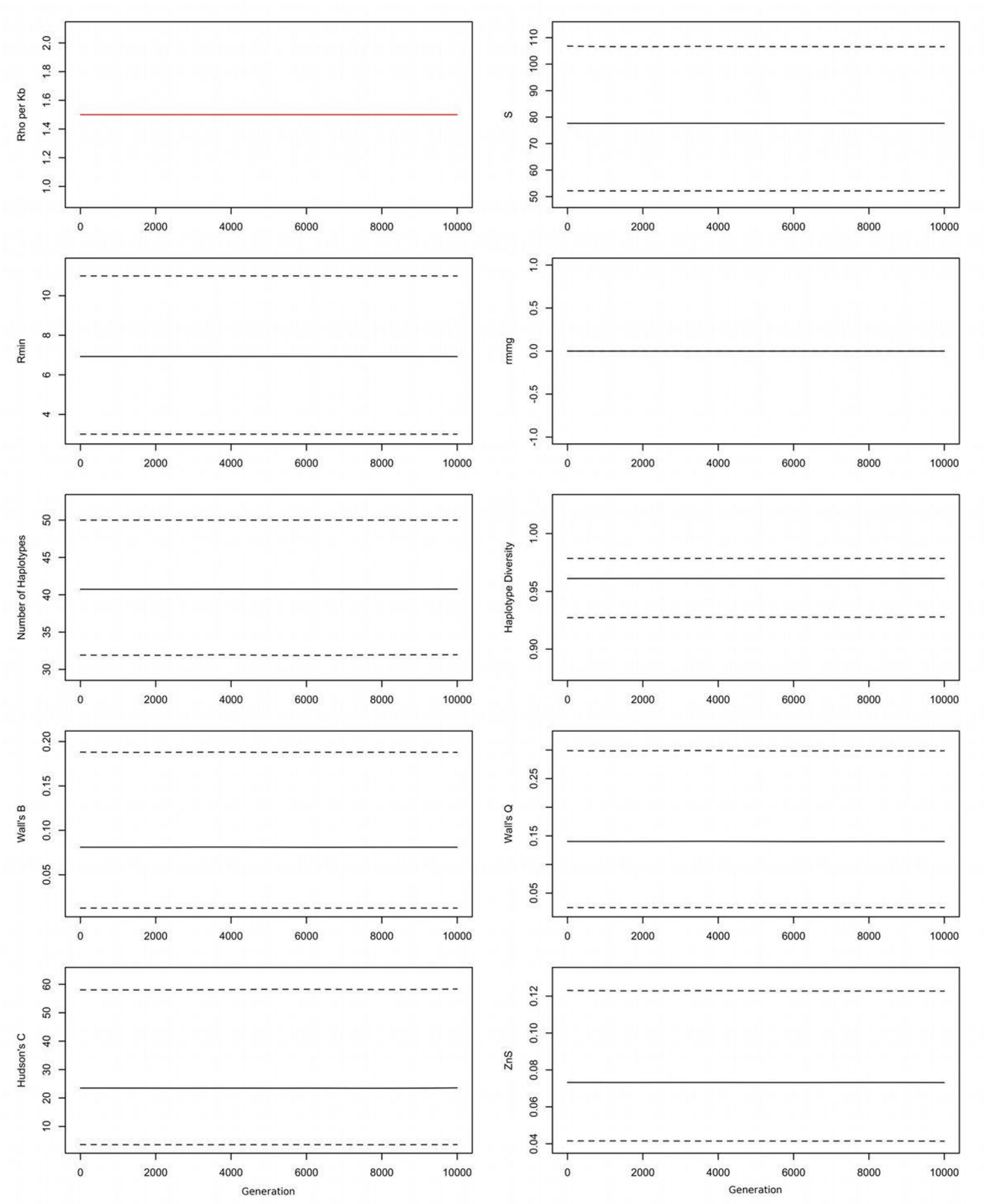

Additional Figure 1 Response of summary statistics to constant recombination rates (ρ per kb) traced over 10^4 generations. Black lines indicate mean (solid) and 95% confidence intervals (dotted) of summary statistic values.
