# Supplementary material for: Reconstructing past changes in locus-specific recombination rates
Source: BMC Genet. 2013 Feb 25;14:11. doi: 10.1186/1471-2156-14-11 (PMC3605148; doi:10.1186/1471-2156-14-11)
Supplement: Additional 3: Figure S3 — Response of summary statistics to recombination rates increasing linearly. [file 1471-2156-14-11-S3.pdf]

Additional Figure 3

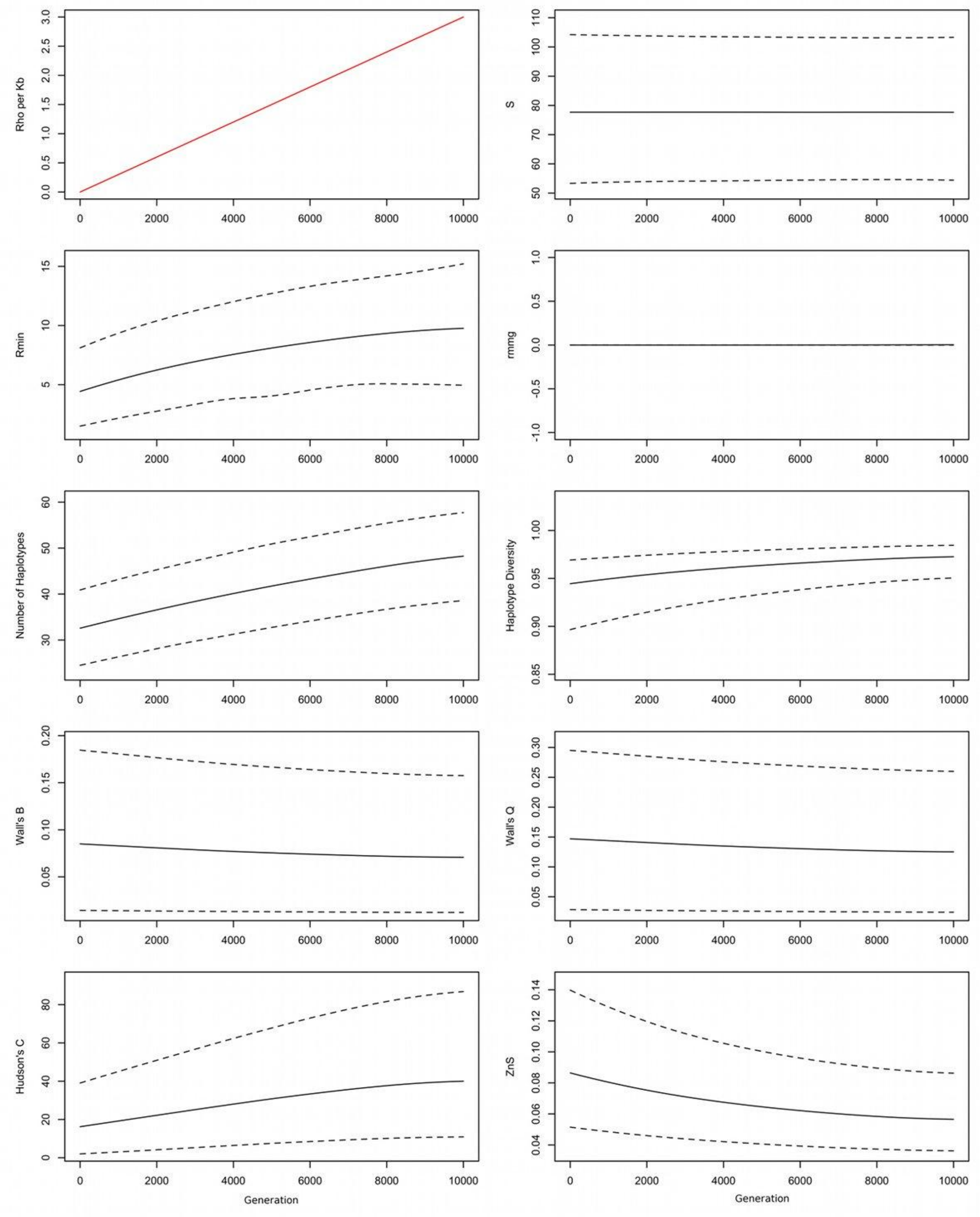

Additional Figure 3 Response of summary statistics to recombination rates (ρ per kb) increasing linearly into the past over 10^4 generations. Black lines indicate mean (solid) and 95% confidence intervals (dotted) of summary statistic values.
