# Supplementary material for: Reconstructing past changes in locus-specific recombination rates
Source: BMC Genet. 2013 Feb 25;14:11. doi: 10.1186/1471-2156-14-11 (PMC3605148; doi:10.1186/1471-2156-14-11)
Supplement: Additional 4: Figure S4 — Response of summary statistics to recombination rates decreasing exponentially. [file 1471-2156-14-11-S4.pdf]

Additional Figure 4

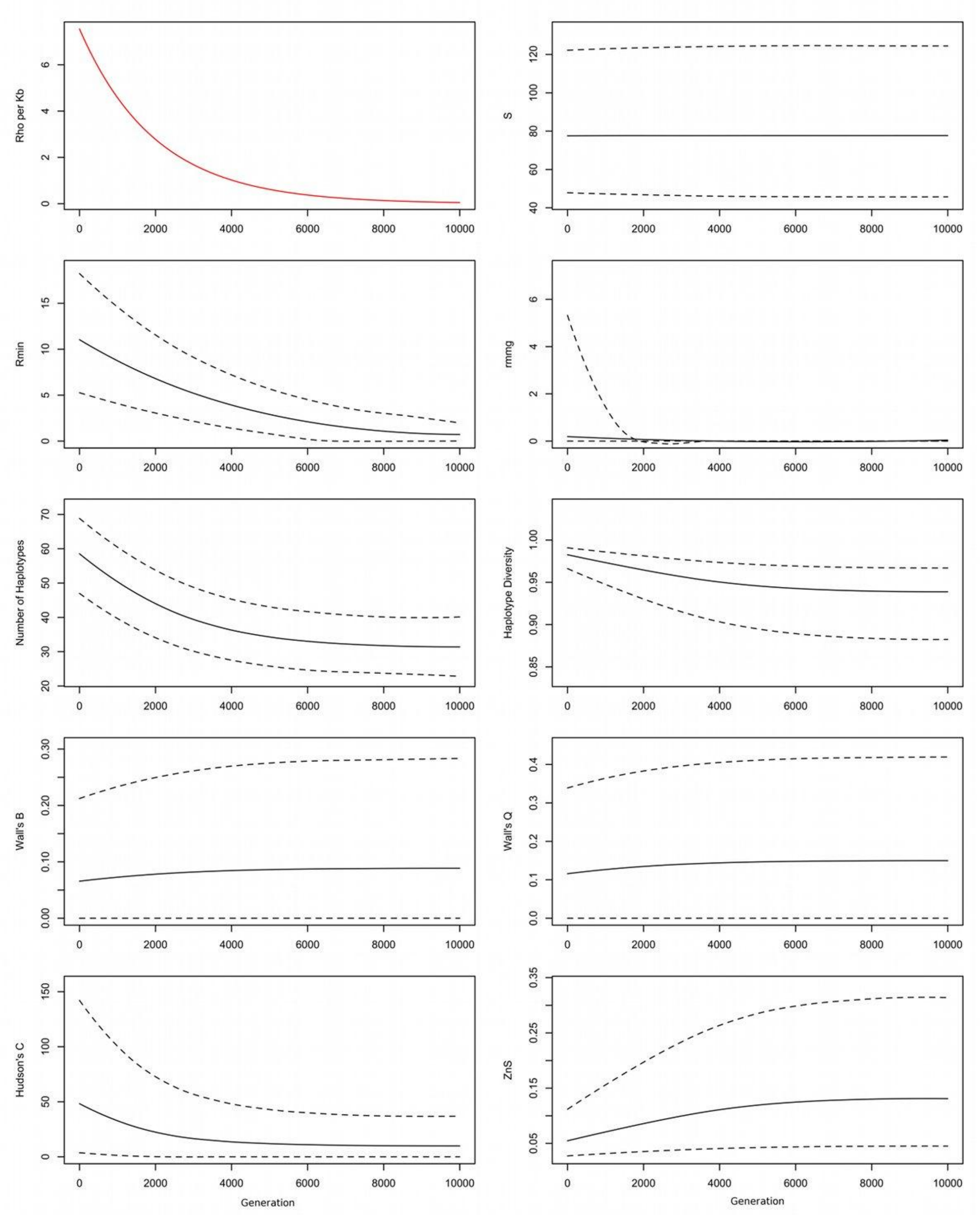

Additional Figure 4 Response of summary statistics to recombination rates (ρ per kb) decreasing exponentially into the past over 10^4 generations. Black lines indicate mean (solid) and 95% confidence intervals (dotted) of summary statistic values.
