# Supplementary material for: Reconstructing past changes in locus-specific recombination rates
Source: BMC Genet. 2013 Feb 25;14:11. doi: 10.1186/1471-2156-14-11 (PMC3605148; doi:10.1186/1471-2156-14-11)
Supplement: Additional 5: Figure S5 — Response of summary statistics to recombination rates increasing exponentially. [file 1471-2156-14-11-S5.pdf]

**Additional Figure 5**

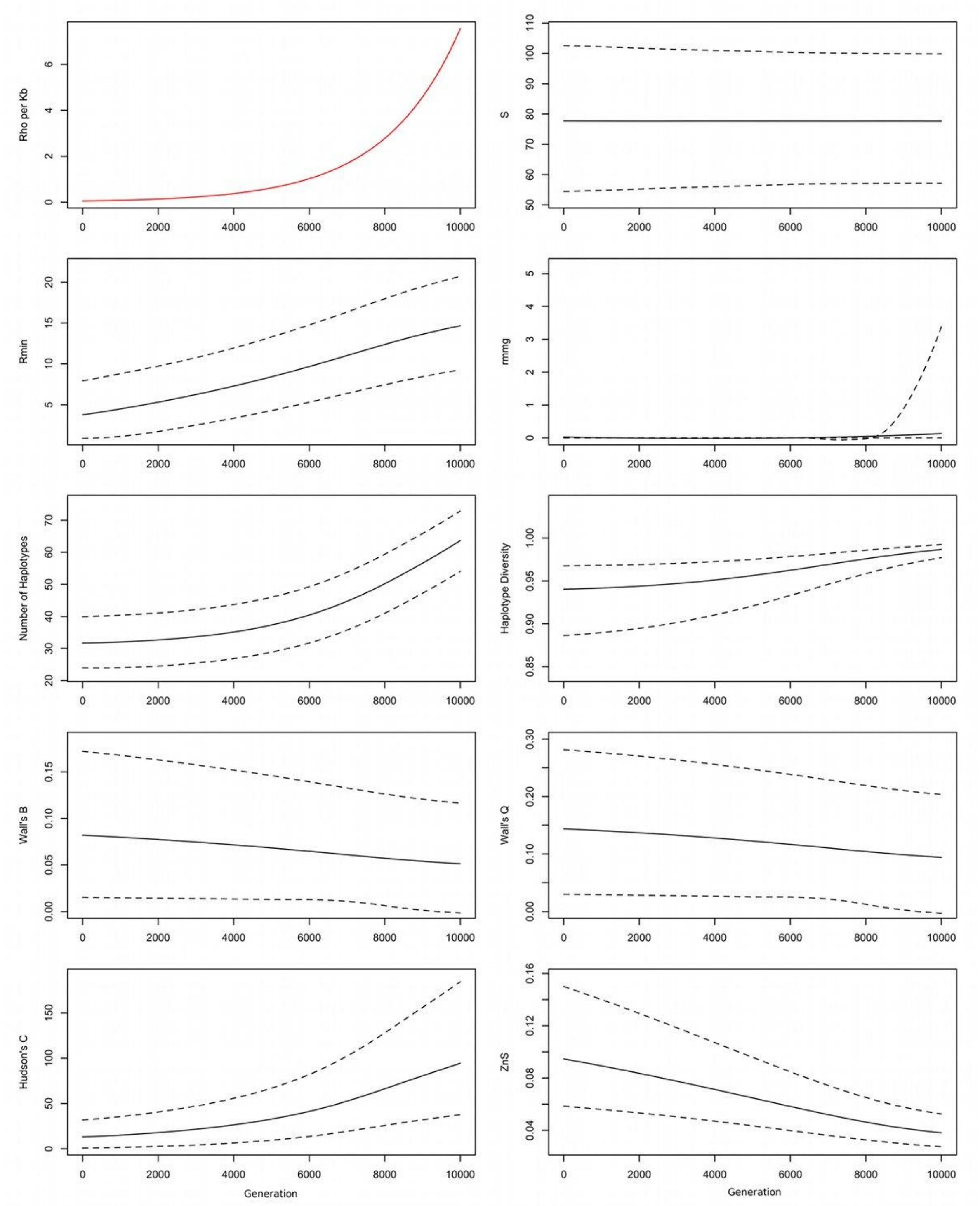


**Additional Figure 5 Response of summary statistics to recombination rates ($\rho$ per kb) increasing exponentially into the past over $10^4$ generations.** Black lines indicate mean (solid) and 95% confidence intervals (dotted) of summary statistic values.
