# Supplementary material for: Reconstructing past changes in locus-specific recombination rates
Source: BMC Genet. 2013 Feb 25;14:11. doi: 10.1186/1471-2156-14-11 (PMC3605148; doi:10.1186/1471-2156-14-11)
Supplement: Additional 8 — Determining the optimal scaling factor to capture n-tuple age. [file 1471-2156-14-11-S8.pdf]

Additional Methods 1

Determining the Optimal Scaling Factor to Capture *n*-Tuple Age

We explored three summaries as potential proxies for the age of each quartet. First, the number of segregating sites *S*, which is an unbiased estimator of the total length of the ancestral recombination graph, and therefore represents the total time elapsed across all branches of the genealogy [1]. Second, the average pairwise distance π, which represents the average coalescent time [2]. Third, the minimum distance between individuals $\min(d_{ij})$, which represents the time to the most recent branching in the genealogy. To our knowledge, $\min(d_{ij})$ has not previously been described, but it has an intuitive interpretation in the quartet setting as being proportional to the amount of time during which four lineages have been present. As some of the recombination statistics are based on detecting non-treelike signal in the data (e.g., the four gametes test of R_{min}, which requires the presence of at least four lineages), we expect that $\min(d_{ij})$ should have greater power than *S* for *n*-tuples with small *n*. For a constant sized population as modeled here, *S* and π are highly correlated [3]. Simulations indicate that *S* and $\min(d_{ij})$ are comparable for small subsample sizes ($4 \le n \le 6$), but the performance of $\min(d_{ij})$ declines quickly with larger subsamples in contrast to *S*. We therefore use only *S* as a scaling factor in our analyses, while noting that π and $\min(d_{ij})$ may perform better for other applications.

1. Watterson GA: **On the number of segregating sites in genetical models without recombination**. *Theor Popul Biol* 1975, **7**(2):256-276.

2. Tajima F: **Evolutionary relationship of DNA sequences in finite populations**. *Genetics* 1983, **105**:437-460.

3. Tajima F: **Statistical method for testing the neutral mutation hypothesis by DNA polymorphism**. *Genetics* 1989, **123**:585-595.
