# Supplementary material for: Reconstructing past changes in locus-specific recombination rates
Source: BMC Genet. 2013 Feb 25;14:11. doi: 10.1186/1471-2156-14-11 (PMC3605148; doi:10.1186/1471-2156-14-11)
Supplement: Additional 9: Figure S8 — Correlations between recombination summary statistics scaled by S. [file 1471-2156-14-11-S9.pdf]

Additional Figure 8

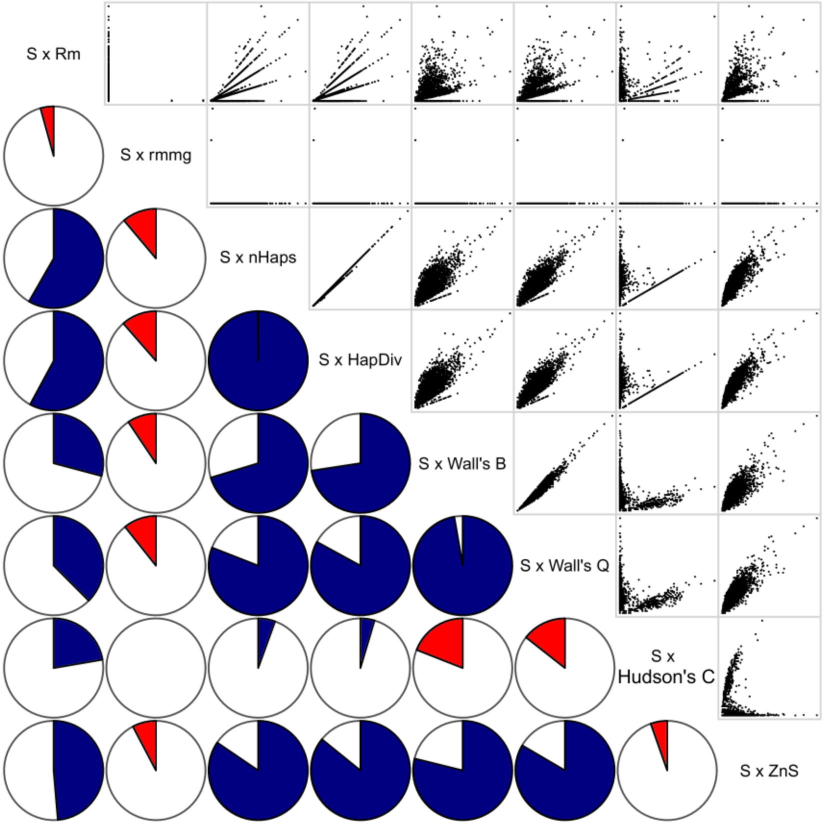

Additional Figure 8 Correlations between recombination summary statistics scaled by *S* for quartet tree length. Pie charts indicate the magnitude of the correlation with blue and red indicating positive and negative values (e.g., Pearson's r = -0.15 for scaled versions of Hudson's C and Z_{nS}).
